# Of Fighting Flies, Mice, and Men: Are Some of the Molecular and Neuronal Mechanisms of Aggression Universal in the Animal Kingdom?

**DOI:** 10.1371/journal.pgen.1005416

**Published:** 2015-08-27

**Authors:** Amanda L. Thomas, Shaun M. Davis, Herman A. Dierick

**Affiliations:** 1 Department of Molecular and Human Genetics, Baylor College of Medicine, Houston, Texas, United States of America; 2 Department of Pathology and Immunology, Baylor College of Medicine, Houston, Texas, United States of America; 3 Department of Neuroscience, Baylor College of Medicine, Houston, Texas, United States of America; 4 Program in Developmental Biology, Baylor College of Medicine, Houston, Texas, United States of America; The Wellcome Trust Centre for Human Genetics, University of Oxford, UNITED KINGDOM

## Abstract

Aggressive behavior is widespread in the animal kingdom, but the degree of molecular conservation between distantly related species is still unclear. Recent reports suggest that at least some of the molecular mechanisms underlying this complex behavior in flies show remarkable similarities with such mechanisms in mice and even humans. Surprisingly, some aspects of neuronal control of aggression also show remarkable similarity between these distantly related species. We will review these recent findings, address the evolutionary implications, and discuss the potential impact for our understanding of human diseases characterized by excessive aggression.

## Introduction

Aggression is a complex social behavior present in most, perhaps even all, animal species. Being aggressive benefits animals to compete for valuable resources but is energetically costly and carries the risk of injury, loss of resources, and even death [1–4]. In humans, symptoms of excessive aggressive behavior can be a complicating factor in the treatment of neurological syndromes and diseases [5–8]. Despite the fact that aggression is vital for organismal fitness and is important in human society and disease, much remains to be discovered about the underlying molecular and neuronal mechanisms that drive this behavior. Decades of research in many model systems have identified several signaling molecules implicated in aggressive behavior, including classical neurotransmitters and neuropeptides, and much has been learned about the brain regions involved in aggression (for review, see [9–11]). However, the degree to which mechanisms in distantly related species are conserved remains largely unexplored.

In this review, we will focus exclusively on recent findings on transcriptional regulation and neuropeptide signaling [12,13] in aggressive behavior in *Drosophila melanogaster*, which suggest an unexpected degree of conservation with mammals. We will speculate on the evolutionary implications of these findings in the vinegar fly as well as their potential implications for human health and disease. For a more in-depth review of the different mechanisms that have been described in different species, several recent reviews provide excellent background that both complement and contrast with the viewpoint that we will discuss here [9–11,14–18].

## Conserved Transcriptional Control of Aggressive Behavior?

**Fig 1:**
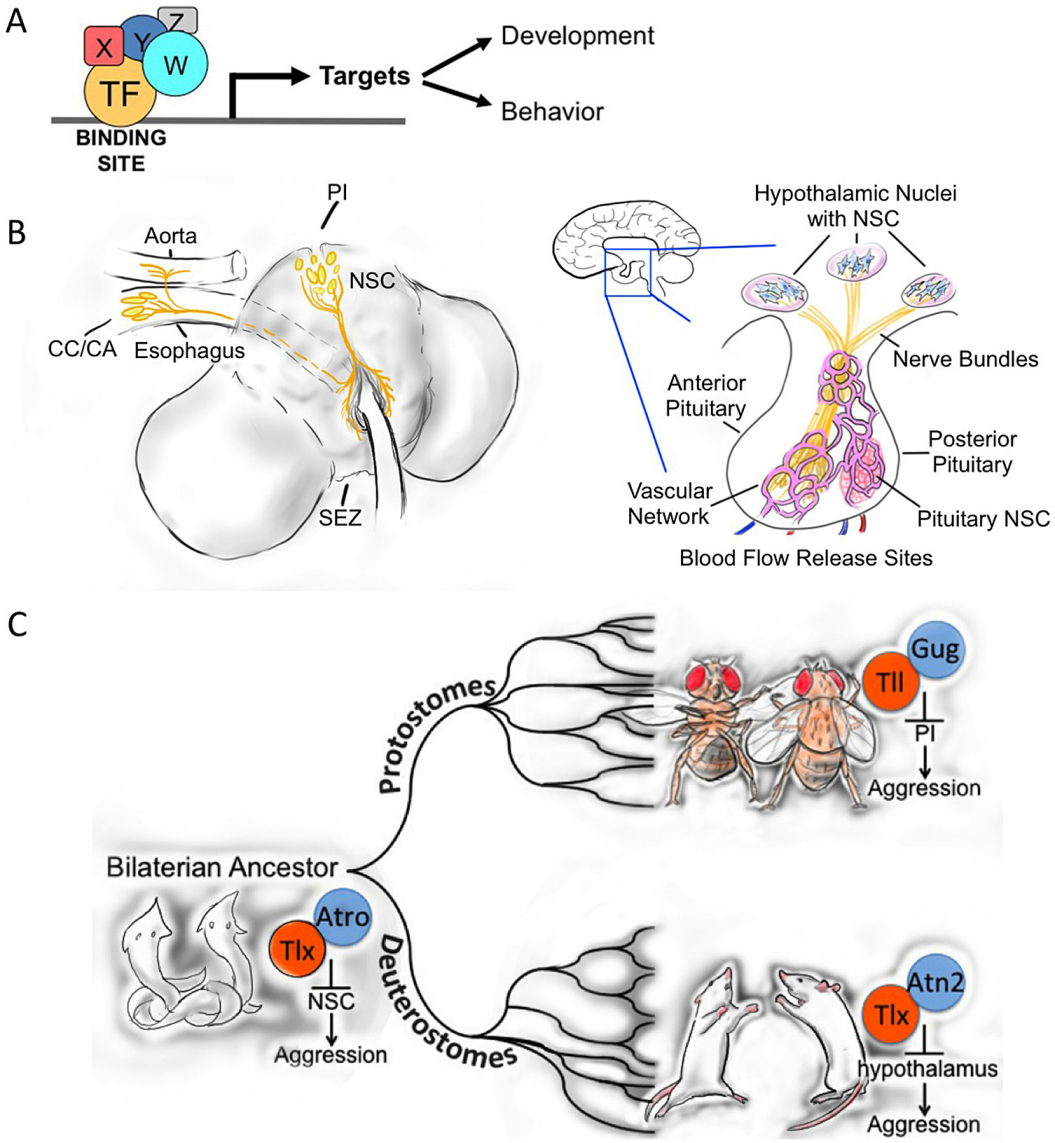
Evolutionary conservation of molecular and neuronal aggression mechanisms. A. Transcriptional control module, consisting of conserved transcription factors, co-factors, DNA binding sites, and activated or repressed target genes, regulating development and/or behavior. B. Comparative diagram of the structural relationship of the *Drosophila PI* and mammalian hypothalamus. C. Simplified evolutionary tree showing the putative conserved transcriptional control mechanism that regulates the release of neuropeptides from the neurosecretory cells in the brain in the regulation of aggression. We postulate that this control module was already present in the bilaterian ancestor (more than 600 million years ago [MYA]) and operates today in extant protostome and deuterostome species (Illustrations courtesy of Josh Rivera). Abbreviations: *PI*, *pars intercerebralis*; NSC, neurosecretory cells; SEZ, subesophageal zone; *CC*, *corpora cardiaca*; *CA*, *corpora allata*.

Conserved transcriptional modules that consist of conserved transcription factors, their co-activators or repressors, DNA binding sites, and targets (Fig 1A) regulate related complex biological programs in organisms throughout the evolutionary tree. The field of developmental biology is replete with examples of conserved transcriptional regulatory mechanisms, although there is also enormous flexibility in the evolutionary deployment of these signaling modules [19–26]. Fewer examples exist in the control of behavior, but the classical example is the conserved transcriptional double feedback loop that regulates circadian rhythm across a broad range of species [27–29]. Konopka isolated several mutations in one of the key transcription factors in this feedback loop in the now-classic screen for altered eclosion rhythm in *Drosophila* [30,31]. This single experiment transformed the field of circadian biology [32], but the idea that a mutation in a single gene could significantly impact “complex” behavior was not without skeptics [33].

Does a conserved transcriptional module that controls aggression in all animals exist? Twenty years ago, a transcriptional repressor orthologous to *Drosophila* Tailless (Tll) was identified in the mouse and shown to be expressed in the developing brain [34]. The mouse knock-out of *tailless* (*tlx*, now called *Nr2e1*) displayed abnormal brain development and behavior, including extreme aggression [35,36]. An independently isolated *Nr2e1* null mutant was called *fierce* because of its extreme pathological aggression [37]. The *fierce* mutant was fully rescued with a genomic clone that covered the human *NR2E1* gene, demonstrating conservation of gene function and regulation between mouse and human [38]. How Nr2e1 regulates aggression, however, has remained unsolved.

Because Tll is a *Drosophila* ortholog of Nr2e1 with conserved co-repressors [39–42], binding sites [34], and targets [43], we explored whether Tll might also affect aggression in *Drosophila*. We found that knock-down of *tll* affects aggression, similarly to its effect in mice. Interestingly, we showed Tll functions through the neurosecretory cells of the adult *pars intercerebralis* (*PI*) [13], a brain region similar to the mammalian hypothalamus [44], which is known for its critical role in aggression [18,45–48].

In mice, *Nr2e1* is expressed in numerous regions throughout the brain, and *Nr2e1- /*- mice have brain abnormalities including reduced brain size, smaller olfactory bulbs, incomplete extension of the cortex, and reduced cortical layers II and III [35,36,49]. It is not clear whether these morphological brain abnormalities cause the behavioral phenotypes, or whether the developmental and behavioral abnormalities can be uncoupled, as is the case in the fly. A conditional mutant was generated using a CamKIIα- Cre line expressing Cre recombinase in the hippocampus, cortex, amygdala, striatum, thalamus, and hypothalamus [50]. Morphologically, these conditional null mutants (*CamKIIα-Cre; Nr2e1Flox/Flox*) have structural brain abnormalities similar to mice derived from a germline mutation in *Nr2e1*, and although they have normal contextual, associative, and spatial learning, they are hyperaggressive, suggesting that a role of Nr2e1 in the regulation of aggressive behavior can be mapped to CamKIIα positive neurons [51]. Whether the effect of Nr2e1 on aggression involves the hypothalamus specifically and whether its effect is developmental or adult specific is currently not known.

Additional evidence for the role of Tll as part of a conserved transcriptional module controlling aggression in *Drosophila* comes from Atrophin, a conserved co-repressor of Tll in flies and mice [40]. Although Atrophin is pan-neuronally expressed, knock-down in the *PI* alone mimicked Tll knock-down in these cells. Moreover, Tll and Atro genetically and physically interact in the *PI*, suggesting that they function together to control aggression through these neurosecretory cells [13].

In mice, two Atrophin paralogs exist, Atrophin 1 (Atn1) and Atrophin 2 (Atn2), each having features in common with the fly ortholog [40]. *Drosophila* Atrophin (also known as Grunge, Gug) shares the most sequence similarity with Atn2 (also known as Rere), which is the longer of the two mammalian Atrophins, but also harbors a polyQ stretch unique to Atn1 [40]. Intriguingly, a polyQ expansion of human ATN1 causes dentatorubral pallidoluysian atrophy (DRPLA) [52,53], a neurodegenerative disease similar to Huntington disease. Patients with this devastating progressive neurodegenerative disorder also have psychiatric symptoms, including abnormal aggressive behavior [54]. In some cases, abnormal behavior precedes the onset of neurodegenerative symptoms [55]. While this could be explained by abnormal neuronal function preceding the actual degeneration of the neurons, it is also possible that the mechanisms that cause neurodegeneration may be uncoupled from the behavioral effects. Interestingly, polyQ expanded Atn1 binds more strongly to Atn2 [56], raising the possibility that the mechanism underlying abnormal aggression in DRPLA patients may depend on diminished ATN2 function and may be part of a putative transcriptional module controlling the function of the hypothalamus, similar to what we found in *Drosophila*. This hypothesis is consistent with the fact that some of the phenotypes associated with polyQ expanded repeats are due to gain-of-function effects, while others are caused by loss-of-function effects [57].

What is the neuronal mechanism of this transcription factor complex in the control of aggression? Given that Tll and Atro function in the *PI* of the adult fly, the neurosecretory function of this brain region is a logical candidate. Blocking neuropeptide processing or release fully suppressed the aggression phenotype induced by knock-down of *tll* [13], showing that neuropeptide signaling is required to mediate the transcriptional regulation of Tll in its control of aggression through the *PI*.

## Conserved Neuropeptide Signaling in the Control of Aggressive Behavior

Neuropeptides are typically involved in the regulation of a specific subset of neurons, as opposed to globally across the entire brain [58]. They play important roles in the control of a range of physiological processes [59] as well as innate behaviors [60]. Several neuropeptides have been implicated in mammalian aggression [61,62], and vasopressin may be the most notable of these [63].

In *Drosophila*, direct evidence for a specific neuropeptide affecting aggression was lacking until recently. Asahina et al. found this evidence by exploring the role of neuropeptides in aggression in a somewhat unusual manner [12]. They cloned parts of the promoters of 18 of the 40 or so known neuropeptide encoding genes in *Drosophila* and used them to transgenically activate the neurons defined by these promoter fragments via a thermosensitive ion channel, TrpA1 [64]. This is one of the many tools developed in flies to manipulate neuronal activity [65], in this case, in a temperature-sensitive manner. They found that the promoter fragment of *Tachykinin*, a *Drosophila* ortholog of *Substance P*, caused a strong aggression response when combined with TrpA1 [12]. Loss of the Tachykinin neuropeptide partially suppressed this response, while its overexpression enhanced it, showing directly that Tachykinin plays a pivotal, albeit not exclusive, role in this response. Interestingly, loss of one of two known *Drosophila* Tachykinin receptor-encoding genes suppressed the response more strongly than loss of the peptide gene itself, indicating that this neuropeptide circuit is indeed important for aggression in flies but may receive other inputs as well. In addition, they found that a few of these Tachykinin-producing neurons are unique to *Drosophila* males, and that activating just this very small number of male-specific peptidergic neurons was necessary and sufficient to elicit the strong behavioral response. As is the case in many species, male flies show dramatically more aggression than females, and these male-specific Tachykinin neurons appear to be critical to this sex difference in aggressive behavior in flies. Although sex determination shows remarkable evolutionary plasticity, even in relatively related species [66,67], it is nevertheless intriguing that *Drosophila* Tachykinin plays a role in aggression because it is related to mammalian Substance P, which has also been implicated in aggression in mammals [68–70] but is probably better known for its role in pain transmission [59]. Even in humans, the levels of Substance P are correlated with aggression levels in certain patient groups [71]. While the evidence in humans for a role of substance P in aggression is limited, together, these results do suggest that another molecular pathway regulating aggression in flies shows conservation with mechanisms in mammals.

The peptidergic neurons defined by the *Tachykinin* promoter are distinct from the neurosecretory cells in the *PI* that are controlled by the action of the Tll/Atro transcriptional repressor complex [12,13]. Activation of the *PI* neurons also showed a significant increase in aggression, and this response was also completely suppressed by blocking neuropeptide processing, showing that activation of these neurons affects their peptidergic function [13]. Multiple neuropeptides are expressed in this complex group of neurons, and it is not clear at present what the culprit neuropeptide is for the aggression response caused by genetic electrical activation of the *PI* neurons or knock-down of *tll* or *Atro*. However, given that Tachykinin is expressed in a separate set of neurons, it suggests that different neuropeptides are involved in the regulation of aggression in flies (and perhaps converging onto the same output system). This complexity also mimics the complex neuropeptidergic control of aggression in mammals [63,69,72]. Such complex neuromodulatory control is also observed in other behaviors in flies and mice, including sleep, feeding, reproduction, and learning and memory [73–78].

## Small Subsets of Neurons with Big Impact on Aggression: Conserved Neuronal Control of Aggression

As is clear from the data presented above, the very few male-specific Tachykinin-producing neurons and the small number of neurosecretory cells in the *PI* have a big impact on aggressive behavior in flies [12,13]. Intriguingly, the *PI* is thought to be very similar to the mammalian hypothalamus [44,79], a structure that is also important in aggression in many different mammals, including humans [18]. The interesting parallelism between the neuroendocrine systems of the *pars intercerebralis*–*corpora cardiaca* (and *allata*) in insects and hypothalamo-pituitary axis in mammals was noted long ago [80]. More recently, it has been argued that the fundamental elements of a primordial neuroendocrine system must have been present in the last common ancestor of flies and mammals, the first bilaterally symmetrical animals, or so-called bilaterian ancestor. This argument is based on structural, developmental, and functional similarities between the *PI* and the mammalian hypothalamus (reviewed in [44]). Other brain structures in insects have been found to show remarkable similarities with structures in the mammalian brain [81–83]. Structural, developmental, and functional similarities in central complex and basal ganglia have been suggested to reflect true homology rather than convergence [82].

Structurally, both the *PI* and hypothalamus contain neurosecretory cells that innervate endocrine centers important in the regulation of growth, metabolism, and reproduction and fertility of the animal (Fig 1B) [84,85]. While the mammalian pituitary combines a posterior neural portion with an anterior glandular portion, these “neuroglandular” parts are separated in insects in the *corpora cardiaca* and *allata*, although these structures are tightly connected in the ring gland of the fly [44,60]. Developmentally, the key signaling molecules that specify the precursor structures that will form the adult neuroendocrine systems in flies and mice are also remarkably conserved [44]. Functionally, the neurosecretory cells of the *PI* and hypothalamus produce a range of neuropeptides and regulate metabolism, growth, water homeostasis, sleep, and feeding behavior [86–94]. We have now shown that the *PI* also affects aggressive behavior in flies [13]. Moreover, in crickets, increased levels of the immediate early marker c-Fos have been detected after crickets engaged in aggressive encounters and after electrical stimulation of the antennae [95].

The evidence for the role of the mammalian hypothalamus in aggression is abundant and has been documented in many species. Studies in small mammals show that activation of specific regions of the hypothalamus is sufficient to initiate aggressive behavior [18,96–98]. In cats and rats, regions of the brain in and around the hypothalamus that control offensive and defensive aggression are known as the hypothalamic attack area [18,48,97]. In non-human primates, electrical stimulation and lesion studies have shown that the hypothalamus plays a role in aggressive vocalizations and physical aggressiveness [99,100]. In humans, abnormal hypothalamic oscillations have been linked to pathologic aggressive behavior [101]. Surgical interventions for hyperaggressiveness have targeted the hypothalamus and successfully reduced aggression in a subset of treated individuals [102–104]. Deep brain stimulation of the posterior hypothalamus has been used to treat hyperaggressive behavior [105,106]. Intraoperative electrical stimulation of a small region in the hypothalamus caused severe transient aggression in previously non-aggressive patients [107]. And a subset of patients with hypothalamic hamartomas display excessive aggressive behavior [108] that is resolved with removal of the tumor [109,110]. These data demonstrate that disruptions in hypothalamic function greatly alter aggressive behavior.

## Evolutionary Implications: Is the Molecular and Neuronal Control of Aggression Deeply Conserved?

The evidence presented above for conserved molecular components that play a role in the control of aggression in flies and mice suggests a deep molecular root for these control mechanisms. The fact that the major neurosecretory regions of the adult brain in flies and mammals are also important in the control of aggression would at first seem quite remarkable, but given the already firm functional connection between the *pars intercerebralis* and the hypothalamus, it should not be that surprising. In *Drosophila*, we connected some of the pieces of the complex puzzle of the regulation of aggression and showed that Tll is critical for the transcriptional control of aggression by regulating neuropeptide release from these neurosecretory centers of the adult fly brain [13]. We propose that Nr2e1 may play the same role in the control of mammalian aggression by regulating hypothalamic release of specific neuropeptides. We speculate that these molecular and neuronal control mechanisms evolved in both protostomes and deuterstomes because the fundamental elements were already present in their last common precursor, the eubilaterian ancestor (Fig 1C). If this were indeed the case, we would expect to find in a very large part of the animal kingdom this unified mechanism of transcriptional control of neuropeptide release from neurosecretory cells in the control of aggression (Fig 1C).

What is the evidence that all these fundamental elements were already present in the last common ancestor of protostomes and deuterostomes? As has been alluded to above and detailed elsewhere [44], neurosecretory cells were already present in these early ancestors, but what about behavior? The urbilaterian and the later eubilaterian ancestors are thought to have been a flatworm-like animal [111–113]. While flatworms occupy a range of phyla [112], there is strong evidence that worm species do indeed show aggressive behavior [114,115]. The marine flatworm, *Pseudoceros bifurcus*, shows a remarkable mating behavior that has been described as “penis fencing” [114] that can last for several hours. When two of these hermaphrodites meet in shallow marine waters they will literally stab each other with their array of penises to inseminate the opponent. The inseminated animal then bears the cost of wound healing and fertilization [114]. Additional evidence that worms fight, despite their simple anatomy and lack of weaponry, was recently shown in the roundworm, *Steinernema longicaudum* (although they belong to the Ecdysozoa-like flies). Under certain conditions, males of this nematode species kill each other by what looks like strangulation [115]. Clearly, worm species are capable of fierce forms of aggression. The urbilaterian ancestor can be believed to have been a fighter.

What is known about the role for their Tailless ortholog? Both roundworms and flatworms have a Tll/Nr2e1 ortholog. In *C*. *elegans* the Tll ortholog is called Nhr67 and plays a number of developmental roles including gustatory neuron specification [116], although nothing is known about its role in behavior. The sequenced genome of the freshwater planaria, *Schmidtea mediterranea*, clearly shows a Tll and Atro ortholog [117]. Knock-down of its *tll*/*Nr2e1* ortholog caused morphological and behavioral defects—shrinking of the brain region and slower movement [118]. Neuropeptide signaling also has deep molecular roots [119]. The idea that a universal transcriptional control module regulates neuropeptide release from adult neurosecretory cells in the control of aggression in all animals is at least plausible and certainly testable. The shared role in aggression of Substance P and a *Drosophila* ortholog, Tachykinin, further support conservation of molecular mechanisms between members of these distantly related phyla.

## Do Any of These Mechanisms Apply to Humans and Human Disease?

As mentioned before, there is a large body of evidence for a role of the hypothalamus in human aggression. Some neuropeptides have also been implicated in human aggression, including Substance P [71]. Human NR2E1 has been associated with bipolar disease, schizophrenia, and psychopathy, diseases known for their association with abnormal aggression, but the evidence for NR2E1 directly affecting aggression in humans is certainly limited. Abnormal aggression has been more strongly connected to DRPLA, the previously mentioned neurodegenerative disease caused by a polyQ expanded repeat in ATN1 [52,53], an ortholog of *Drosophila* Atrophin [40]. A mouse model for this disease recapitulates both neurodegenerative phenotypes and excessive aggression [120,121] but, as mentioned above, the mechanism is not currently known.

**Fig 2:**
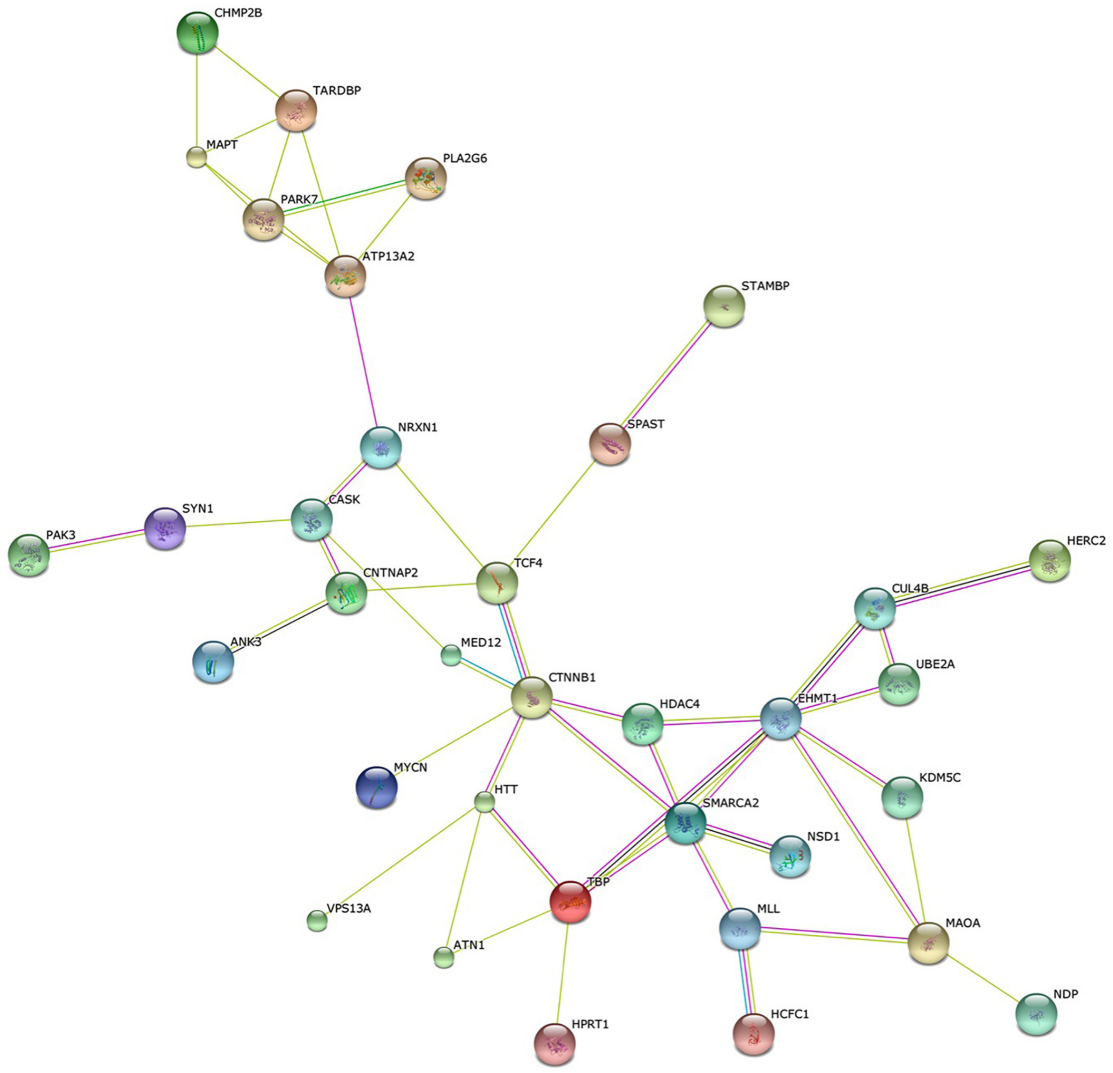
Human aggression disease interactome. String analysis of a subset of proteins encoded by disease genes that are characterized by excessive aggression as part of their clinical picture. Roughly 40% of the approximately 90 genes in this category in OMIM form a single network (*p* = 4.7–10, S1 Table), suggesting that there are mechanistic connections between these components.

To take a closer look at the connection between human disease and aggression, we compiled a list of all known Mendelian disorders with excessive aggressive behavior as part of their clinical picture. This search in Online Mendelian Inheritance in Man (OMIM) [122] produces a list of approximately 90 diseases and their causal genes. Although many of these diseases are developmental disorders characterized by mental retardation, intellectual disability alone is not likely to account for the increased aggression symptoms since there are more than 400 genes that cause intellectual disability [123] and relatively few are associated with increased aggression and some are associated with pleasant behavior [124–126]. Almost half of the proteins encoded by these disease genes are connected into a single network based on STRING analysis (Fig 2 and S1 Table) [127]. Not all of the interactions represent direct protein—protein interactions, but many of them do. The small number of disease genes associated with aggression and the highly connected nature of their network suggests specific and finite mechanisms. This observation stands in stark contrast with the suggestion that one-third of the genome may play a significant role in aggressive behavior [16]. One simple explanation for this discrepancy may be the low specificity of their assay used to assess aggression in flies. Although we still know very little about the regulation of aggression in humans and although it is no doubt more complex than in mice, let alone flies, investigating the role of this protein network in the control of the peptidergic function of the hypothalamus would be a good place to start.

## Conclusion

Aggression is pervasive throughout the animal kingdom and is both a beneficial and costly behavior. Recent evidence suggests that both molecular and neuronal mechanisms underlying aggressive behavior may be conserved throughout evolutionary history. The orphan nuclear receptor Nr2e1 and its associated co-repressors affect aggressive behavior in both flies and mice, and evidence in flies shows that its effect on aggressive behavior depends on the neurosecretory function of a very small number of neurons in the adult brain. In humans, NR2E1 is part of a large protein interactome of disease genes with a clinical picture marked by aggression, suggesting that this transcriptional module may be part of a larger network of proteins that also regulate aggression in humans. Further examination of the role of this conserved transcriptional module should be carried out to better understand its role in aggression in animal models, as well as to pursue a putative explanation for some of the heterogeneity observed in human populations and disease.

## Supporting Information

S1 Table Diseases associated with excessive aggression and their causative genes shown in the network in Fig 2. (DOCX)
