# Supplementary material for: Of Fighting Flies, Mice, and Men: Are Some of the Molecular and Neuronal Mechanisms of Aggression Universal in the Animal Kingdom?
Source: PLoS Genet. 2015 Aug 27;11(8):e1005416. doi: 10.1371/journal.pgen.1005416 (PMC4551476; doi:10.1371/journal.pgen.1005416)
Supplement: S1 Table — (DOCX) [file pgen.1005416.s001.docx]

Table S1 Diseases associated with excessive aggression and their causative genes shown in the network in Figure 2.

| Disease/Syndrome | Phenotype MIM # | Causative Gene | Locus MIM # |
| --- | --- | --- | --- |
| Frontotemporal Dementia, Chromosome 3-Linked; FTD3 | 600795 | CHMP2B | 609512 |
| Levine-Critchley Syndrome | 200150 | VPS13A | 605978 |
| Parkinson Disease 14, Autosomal Recessive; PARK14 | 612953 | PLA2G6 | 603604 |
| Spinocerebellar Ataxia 17; SCA17 | 607136 | TBP | 600075 |
| Huntington Disease; HD | 143100 | HTT | 613004 |
| Mental Retardation, Autosomal Recessive 37; MRT37 | 615493 | ANK3 | 600465 |
| Kleefstra Syndrome | 610253 | EHMT1 | 607001 |
| Feingold Syndrome | 164280 | MYCN | 164840 |
| Feingold Synfrome 4 | 300422 | CASK | 300172 |
| Brachydactyly-Mental Retardation Syndrome; BDMR | 600430 | HDAC4 | 605314 |
| Hairy Elbows, Short Stature, Facial Dysmorphism, And Developmental Delay, Wiedemann-Steiner Syndrome | 605130 | MLL/KMT2A | 159555 |
| Pitt-Hopkins Syndrome; PTHS | 610954 | TCF4 | 602272 |
| Pitt-Hopkins-Like Syndrome 1, Included; PTHSL1, Included | 610042 | CNTNAP2 | 604569 |
| Pitt-Hopkins-Like Syndrome 2; PTHSL2 | 614325 | NRXN1 | 600565 |
| Dentato-Rubral-Pallidoluysian Atrophy, DRPLA | 125370 | ATN1 | 607462 |
| Brunner Syndrome | 300615 | MAOA | 309850 |
| Mental Retardation Autosomal Dominant, MRD19 | 615075 | CTNNB1 | 116806 |
| Lujan-Fryns Syndrome, Opitz-Kaveggia Syndrome | 309520/305450 | MED12 | 300188 |
| Mental Retardation, X-Linked, Syndromic, Claes-Jensen Type; MRXSCJ | 300534 | KDM5C/JARID1C | 314690 |
| Mental Retardation, X-Linked, Cabezas Type | 300354 | CUL4B | 300304 |
| Epilepsy, X-Linked, With Variable Learning Disabilities And Behavior Disorders | 300491 | SYN1 | 313440 |
| Mental Retardation, X-Linked, Syndromic, Nascimento Type; MRXSN | 300860 | UBE2A | 312180 |
| Nicolaides-Baraitser Syndrome; NCBRS | 601358 | SMARCA2 | 600014 |
| Methylmalonic Acidemia And Homocysteinemia, cblX TYPE | 309541 | HCFC1 | 300019 |
| Mucopolysaccharidosis Type IIIb | 615516 | HERC2 | 605837 |
| Norrie Disease; ND | 310600 | NDP | 300658 |
| Mental Retardation, X-Linked 30; MRX30 | 300558 | PAK3 | 300142 |
| Kufor-Rakeb Syndrome; KRS | 606693 | ATP13A2 | 610513 |
| Spastic Paraplegia 4, Autosomal Dominant; SPG4 | 182601 | SPAST | 604277 |
| Sotos Syndrome | 117550 | NSD1 | 606681 |
| Microcephaly-Capillary Malformation Syndrome; MICCAP | 614261 | STAMBP | 606247 |
| Lesch-Nyhan Syndrome; LNS | 300322 | HPRT1 | 308000 |
| Parkinson Disease 7, Autosomal Recessive Early-Onset; PARK7 | 606323 | DJ1 | 602533 |
| Amyotrophic Lateral Sclerosis 10, With Or Without Frontotemporal Dementia; ALS10 | 612069 | TARDBP | 605078 |
| Pick Disease | 172700 | MAPT | 157140 |
